# Mammals with Small Populations Do Not Exhibit Larger Genomes

**DOI:** 10.1093/molbev/msab142

**Published:** 2021-05-06

**Authors:** Adam B Roddy, David Alvarez-Ponce, Scott W Roy

**Affiliations:** 1 Institute of Environment, Department of Biological Sciences, Florida International University, Miami, FL, USA; 2 Biology Department, University of Nevada, Reno, NV, USA; 3 Department of Biology, San Francisco State University, San Francisco, CA, USA

**Keywords:** evolution of complexity, C-value paradox, population genomics, drift barrier hypothesis, genome complexity

## Abstract

Genome size in cellular organisms varies by six orders of magnitude, yet the cause of this large variation remains unexplained. The influential Drift-Barrier Hypothesis proposes that large genomes tend to evolve in small populations due to inefficient selection. However, to our knowledge no explicit tests of the Drift-Barrier Hypothesis have been reported. We performed the first explicit test, by comparing estimated census population size and genome size in mammals while incorporating potential covariates and the effect of shared evolutionary history. We found a lack of correlation between census population size and genome size among 199 species of mammals. These results suggest that population size is not the predominant factor influencing genome size and that the Drift-Barrier Hypothesis should be considered provisional.

Genome size in cellular organisms varies by six orders of magnitude (Gregory et al. 2007). This variation shows no clear association with organismal complexity and, in general, remains unexplained (Eddy 2012). Genome size can increase due to an array of processes, such as polyploidization, amplification of repetitive DNA (including tandem repeats and transposable elements), gene duplication, and other insertions, the effects of which can be counteracted by DNA loss. Some models of genome size evolution assume that genome size impacts fitness though cell size (Bennett 1971; Gregory and Hebert 1999) or the nuclear/cytosol volume ratio (Cavalier-Smith 1978, 2005) and their effects on phenotypes, such as body size, developmental timing, and metabolic rates (Roddy et al. 2020). Other models assume that most changes in genome size are nearly neutral (Petrov 2002; Lynch and Conery 2003; Lynch 2007; for review, see Blommaert 2020).

Perhaps the most influential modern hypothesis for this variation, the Drift-Barrier Hypothesis (Lynch and Conery 2003; Lynch 2007), proposes a key role for effective population size (*N_e_*). According to this hypothesis, if many mutations that increase genome size are slightly deleterious, such mutations are much more likely to fix under conditions in which stochasticity plays a greater role relative to selection, namely under the low selective efficiency experienced by small populations (or more generally populations with small *N_e_*; Lynch and Conery 2003; Lynch 2007). Thus, the Drift-Barrier Hypothesis predicts a negative correlation between *N_e_* and genome size. Despite the broad influence of the Drift-Barrier Hypothesis, few explicit tests have been reported (Yi and Streelman 2005; Whitney and Garland 2010; Lefébure et al. 2017), largely due to the rarity of data sets with accurate estimates for *N_e_*.

Although *N_e_* is not identical to, and is consistently lower than, census population size (*N_c_*), *N_c_* is expected to be among the most important determinants of *N_e_*. Indeed, various studies have found clear correlations between *N_e_* and *N_c_* (e.g., James and Eyre-Walker 2020). Here, we performed the first explicit test of a relationship between *N_c_* and genome size within a single taxonomic group. We combined data on mammalian genome size from the Animal Genome Size Database (Gregory et al. 2007) with data on estimated *N_c_* (estimated as the product of population density and geographic area from census data) from the PanTheria database (Jones et al. 2009), leading to a total of 199 species with values for both traits (see supplementary Materials and Methods and table S1, Supplementary Material online).

A simple correlation analysis between *N_c_* and genome size showed a nonsignificant positive relationship (that is, opposite to the predicted direction; Spearman’s rank correlation coefficient, ρ = 0.0006, *n *=* *199, *P *=* *0.99), which was consistent with linear regression analysis (*t *=* *0.09, df = 197, *P *=* *0.93) (fig. 1*A*). Concerned about the effects of shared phylogenetic history on this relationship, we performed several additional tests. First, we asked whether genome size and *N_c_* tended to correlate within specific taxonomic ranks (fig. 1*B*; data available in supplementary tables S2–S4, Supplementary Material online). More genera showed a positive correlation (18 genera) than showed the negative correlation predicted by the Drift-Barrier Hypothesis (10 genera), although this difference was not significant (*P *=* *0.18 by a binomial test; supplementary table S2, Supplementary Material online). The same was true when we evaluated correlations within families (22 positive, 10 negative; *P *=* *0.0501; supplementary table S3, Supplementary Material online) and within orders (nine positive, five negative; *P *=* *0.42; supplementary table S4, Supplementary Material online). Second, a systematic correction for phylogeny by using phylogenetically independent contrasts similarly showed a nonsignificant positive correlation (ρ = 0.10, *P *=* *0.15), which was consistent with phylogenetic least squares regression, which also showed a nonsignificant positive effect of *N_c_* on genome size (*t *=* *1.20, df = 197, *P* = 0.23; fig. 1; table 2). Overall, these results suggested that among mammals there is no negative effect of *N_c_* on genome size.

**Fig. 1:**
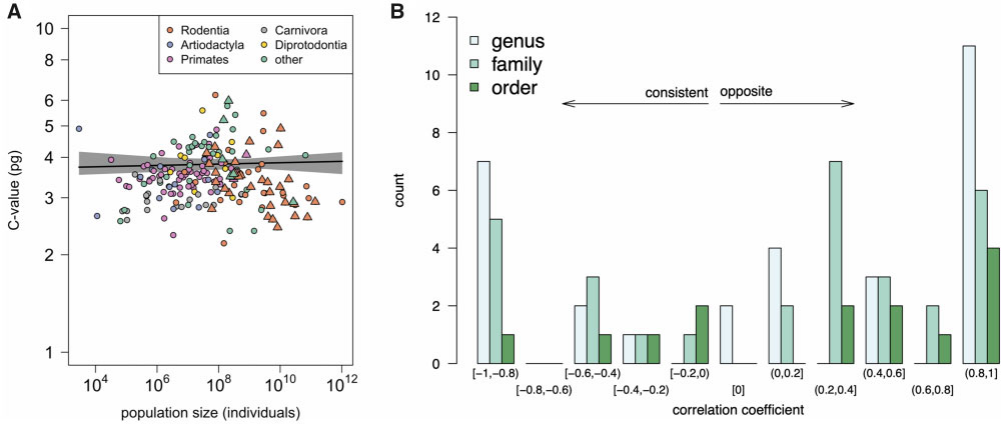
Relationship between genome size (C-value) and population size for mammals. (*A*) No correlation between genome size and population size. Points represent individual species, and points are colored according to their taxonomic order. Point symbols distinguish large- (circles) and small-bodied (triangles) mammals used in the regressions. The solid line and shading represent the mean and confidence intervals for the phylogenetic regression, which was not statistically significant. (*B*) Correlations within taxonomic ranks. For all three ranks, fewer taxonomic groups show a negative correlation (consistent with the prediction) than show a positive correlation (opposite to the prediction).

**Table 2:** Generalized Least Squares Regression Results, with and without Phylogenetic Control.

Model	Nonphylogenetic			Phylogenetic				
	*T*	df	*P*	*t*	df	*P*	Trees with Negative Effect	Trees with Significant Effect
All mammals								
Genome ∼ population	–0.09	197	0 .93	1.2	197	0.23	16/100	0/100
genome ∼ population * mass * metmass	–2.95	65	**0.004**	–0.51	73	0.61	100/100	0/100
genome ∼ population * mass	–4.01	74	**0.000**	–0.59	75	0.56	96/100	0/100
genome ∼ population + mass	–0.07	75	0 .95	2.92	75	**0.005**	0/100	100/100
Body mass < 316 g								
genome ∼ population	–3.31	37	**0.002**	0.7	37	0.49	0/100	0/100
genome ∼ population * mass * metmass	–1.39	29	0 .17	0	37	1	24/100	0/100
genome ∼ population * mass	–1.34	35	0 .19	0.02	39	0.98	0/100	0/100
genome ∼ population + mass	–2.6	36	**0.01**	0.82	39	0.41	0/100	0/100
Rodentia								
genome ∼ population	–2.18	57	**0.03**	1.21	59	0.23	6/100	1/100
genome ∼ population * mass * metmass	–0.63	23	0.53	0.84	31	0.41	0/100	0/100
genome ∼ population * mass	–2.59	29	**0.02**	–0.5	33	0.62	100/100	0/100
genome ∼ population + mass	–0.78	30	0.44	1.36	33	0.18	0/100	0/100

Note.—Regressions incorporating phylogenetic covariation were repeated across 100 randomly chosen, equally likely trees (see supplementary information, Supplementary Material online), and the test statistics reported are from one randomly chosen tree. The last two columns indicate for how many of these 100 trees the effect of population size was negative (consistent with the prediction) and for how many of these trees the effect of population size was significant (with either positive or negative effect or population size). Test statistics are for the effect of population size, after accounting for other variables in the model, and the sign of the *t* statistic indicates the sign of the slope for population size.

Other traits, such as body size and metabolic rate, are known to correlate with both *N_c_* and genome size and could mediate the relationship between genome size and *N_c_* (Vinogradov 1995). Consistent with prior analyses, we observed similar relationships: body size and *N_c_* were negatively correlated (*r*^2^ = 0.48, *P *<* *0.0001), body size and genome size were positively correlated (*r*^2^ = 0.12, *P *<* *0.0001), basal metabolic rate and genome size were positively correlated (*r*^2^ = 0.08, *P *<* *0.0001), and metabolism per unit body mass and genome size were negatively correlated (*r*^2^ = 0.12, *P *<* *0.0001). We therefore regressed both genome size and *N_c_* onto body size and metabolic rate and performed regression analyses on the residuals, thereby controlling for the effects of body size and metabolic rate (table 1). Whether total basal metabolic rate or basal metabolic rate per unit body mass were used, we found that both total genome size and residual genome size showed nonsignificant- and often positive-correlations with both total *N_c_* and residual *N_c_* (table 1). Thus, even accounting for the variance in *N_c_* and genome size explained by organismal traits (body size and metabolism), there was no relationship between genome size and *N_c_*. We also tested for the effects of *N_c_* on genome size using a generalized least squares framework that incorporated body mass and metabolism, with and without phylogenetic control (table 2). Although some of these models found a statistically significant, negative effect of *N_c_* on genome size without phylogenetic correction, these effects were not significant after accounting for phylogenetic covariation. Indeed, among the various phylogenetically corrected models tested, only one showed a significant result, and this test showed a positive effect, opposite to the prediction (table 2).

We also ran the same tests on two subsets of the data set in an effort to find cases in which the Drift-Barrier Hypothesis may be supported. First, because organisms with small body sizes may be more robust to anthropogenic disturbance (Wan et al. 2019), we repeated these analyses on only species with body mass lower than 316 g (the median body mass among species in the data set). Although a negative effect of *N_c_* on genome size was detected for two of the nonphylogenetic models, these relationships became nonsignificant and changed to being positive when accounting for phylogenetic history (table 2). Second, we focused our analyses solely on the order Rodentia, because they are species-rich, have generally large *N_c_*, and may be more robust to human disturbance. As above, although two nonphylogenetic models showed significant, negative effects of *N_c_* on genome size, these were no longer significant after accounting for shared evolutionary history.

**Table 1:** Generalized Least Squares Regression Results of Residual Variation.

	*t*	df	*P*
genome size ∼ residuals(population size ∼ mass * metabolism)	0.6	71	0.55
population size ∼ residuals(genome size ∼ mass * metabolism)	0.48	71	0.64
residuals(genome size ∼ mass * metabolism) ∼ residuals(population size ∼ mass * metabolism)	–0.485	71	0.63

Although *N_c_* is an important contributor to *N_e_*, and various data confirm a positive correlation between the two (e.g., James and Eyre-Walker 2020), they are certainly not identical. Various factors can affect the *N_e_*/*N_c_* ratio, which are not accounted for in our analyses. Among the factors classically thought to affect *N_e_* are skewed sex ratios, overlapping generations, fluctuating population sizes and population subdivision (e.g., Hartl and Clark 1997). Unfortunately, we lack information on these factors for most of the species included in our analyses. How does our failure to account or these factors affect our analysis?

First, some of these factors are expected to reinforce interspecific differences in *N_c_*. For instance, overlapping of generations, which is expected to decrease *N_e_* relative to *N_c_*, is likely to be more common in long-lived and large-bodied mammals; thus, accounting for the contribution of overlapping generations is expected to exacerbate interspecific differences in *N_c_* and therefore reinforce rather than obscure correlations with *N_e_.* Another consideration is fluctuation in *N_c_*, which is expected to depress *N_e_*. Insofar as such fluctuations are stochastic, this factor may be greater in populations with small *N_c_*, which experience greater stochasticity; thus, as with overlapping generations, the effect of fluctuations on *N_e_* may reinforce rather than obscure interspecific differences in *N_c_*. Variation in reproductive success is also expected to decrease *N_e_* relative to *N_c_*. Variation in reproductive success across males in mammals is often associated with harem societies in which older males dominate mating. If so, this effect may be greater in larger, long-lived mammals, again reinforcing interspecific differences in *N_c_*. To our knowledge, there has been no comparative analysis of variation in reproductive success, which would help clarify the magnitude of this effect.

Second, contributions from such factors are expected to be quite small relative to differences in *N_c_*. For instance, skewed sex ratios are expected to reduce effective population size by a factor 1–4*d*^2^, where *d* is the deviation from equal sex ratios (i.e., proportion of males = 0.5–*d*) (e.g., Hartl and Clark 1997). Based on the largest comparison of mammalian sex ratios of which we are aware (Berger and Gompper 1999), the contribution to the variance in *N_e_* due to estimated sex ratios is 0.004 on a log scale, i.e., small compared to the contribution of *N_c_* (2.33 on a log scale). Moreover, the authors found no correlation with body size, suggesting no relationship with *N_c_*. Similarly, theoretical studies suggest that dispersal has a very small effect on *N_e_*, except in the case of extraordinarily low dispersal (i.e., a neighborhood size <∼12 individuals; Nunney 2016). Similarly, depression of *N_e_* due to inbreeding is unlikely to be a major factor in mammals, which tend to have negative *F*_IS_ values (i.e., a bias towards outbreeding; e.g., Storz 1999).

None of the above should be taken to deny that *N_e_* is depressed relative to census population size, nor that the extent to which it is depressed varies across species. Instead, as is increasingly appreciated, it seems more likely that rates of selection on linked sites are likely to dominate any effects of the *N_e_*/*N* ratio (e.g., Corbett-Detig et al. 2015). However, insofar as such factors as the degree of selection on linked sites are also likely difficult to directly estimate beyond the very recent past, there may be no better metrics of effective population size than silent site diversity, *d*_N_/*d*_S_, and now *N_c_*.

Although our analysis revealed no significant relationship between *N_c_* and genome size in mammals, we cannot conclude that there is no effect of *N_e_* on genome size at all. Rather, our analysis suggests, contrary to the Drift-Barrier Hypothesis, that *N_e_* has, at best, a minor impact on genome size in mammals. There are numerous factors that have been discussed and documented previously, all of which would influence genome size (Blommaert 2020). Neither these alternative factors nor the role of *N_e_* should be ignored. Indeed, although we believe that there is a role for *N_e_* in driving genome size variation, its role, at least for mammals, seems limited compared with that of the various other factors. It would be challenging to reconcile the notion that *N_e_* plays the dominant role in genome size despite all of life’s diversity, and yet has an insufficient impact to be seen in a comparison of 199 species.

Despite the ubiquity of the Drift-Barrier Hypothesis in the literature, our analysis is one of the first explicit tests of the relationship between population size and genome size. Using proxies for population size, such as trophic level, habitat, or rate of protein evolution ( Vinogradov 2004; Yi and Streelman 2005; Lefébure et al. 2017), can confound results with other possible interpretations. For example, the observation that freshwater fish have larger genome sizes than marine fish could reflect smaller population sizes (Yi and Streelman 2005), or it could reflect greater environmental variation in freshwater environments, which is also associated with larger genomes (Smith and Gregory 2009). Similarly, although habitat was used as an a priori proxy for population size in isopods and was found to not correlate with genome size, a post hoc proxy, increased protein evolutionary rate, did correlate with genome size (Lefébure et al. 2017). However, other explanations for this correlation, including general correlations in rate of evolutionary change (i.e., association of rapid protein evolution with rapid change of genome size/structure; Irimia et al. 2012) and increased fixation of deleterious amino acid changes due to background selection on transposable element insertions (Charlesworth 1994), remain to be explored. Furthermore, other work has failed to find a correlation between genome size and two proxies of population size: protein evolutionary rate and degree of polymorphism (Whitney and Garland 2010). One of these studies showed no intron gain in various cases of very low effective population size, contrary to the predictions of the Drift-Barrier Hypothesis (Roy 2016).

The current study represents, to our knowledge, the first comparison of direct estimates of genome size and population size within a single taxonomic group. We have adhered to the approaches suggested by proponents of the Drift-Barrier Hypothesis, both in testing relationships within a single taxonomic group and in eschewing indirect estimates of population size from polymorphism data (Lynch 2011). Although in some cases a significant, negative effect of population size on genome size was detected, this effect repeatedly became statistically nonsignificant after accounting for shared evolutionary history, highlighting that genome size and population size do not evolve together. Despite trying to account for other potentially confounding variables in an explicit attempt to find support for the Drift-Barrier Hypothesis, we repeatedly failed to find statistically robust support. These results suggest that the Drift-Barrier Hypothesis of genome evolution should be regarded, at best, as provisional.

## Supplementary Material

Supplementary data are available at *Molecular Biology and Evolution* online.

## Supplementary Material

msab142_Supplementary_Data
