# Supplementary material for: Mammals with Small Populations Do Not Exhibit Larger Genomes
Source: Mol Biol Evol. 2021 May 6;38(9):3737–41. doi: 10.1093/molbev/msab142 (PMC8382904; doi:10.1093/molbev/msab142)
Supplement: msab142_Supplementary_Data [file msab142_supplementary_data.zip › mammals_supplement.docx]

**Supplemental Information**

*Methods*

*Data access and compilation*

All mammalian genome sizes (C-values) were retrieved from the Animal Genome Size Database (Gregory et al. 2007) in March 2018. For each species, all available genome sizes were averaged. For each species, distribution area and population density were extracted from the PanTheria database (Jones et al. 2009), and population size was estimated as the simple product of distribution area and population density. Only species with genome size and population size data were included in our analyses (*n* = 199). Analyses including body mass and metabolism had fewer species because these variables were not available in PanTheria for all 199 species with genome size and population size.

*Phylogeny*

Using the full species list from the combination of the PanTheria and Animal Genome Size databases, we queried VertLife.org (Upham et al. 2019) for species-level phylogenies. This approach produced 100 trees that incorporated uncertainty in the species-level relationships among mammals. Because these trees were not perfectly ultrametric, we extended the tips of each tree to make them ultrametric. No single branch on any tree had more than 99.2 years in branch length added in order to ultrametricize the tree. Across all trees, the average branch length added to the tips to ultrametricize the tree was 29.03 ± 8.31 years. Compared to the average total height of each tree (188.83 million years), the amount of branch length added was likely inconsequential.

*Statistical analyses*

Both genome size and census population size were log-transformed to improve normality, and all subsequent analyses were performed on log-transformed data. For each genus, family, and order containing data for two or more species in our dataset, we evaluated the correlation between genome size and population size using Spearman’s rank order correlation. These correlations are available in the Supplemental Dataset.

To incorporate multiple covariates (e.g. body mass, metabolic rate), we used generalized least squares regression, as implemented in the R package ‘nlme’. The sample sizes depended on the subsets of the data set analyzed in each test (Table 2). To account for the possible effects of shared phylogenetic history, we performed two types of analyses. First, we calculated phylogenetically independent contrasts (PICs) using the R package ‘ape’ and tested relationships between PICs using Spearman’s rank test correlation. Second, we incorporated share phylogenetic history into the GLS models described above. To do this, we used Brownian motion correlation structure based on the phylogeny, using the *corBrownian* function in the R package ‘ape’. A maximum likelihood approach was used to fit these phylogenetic GLS models. To account for phylogenetic uncertainty in these analyses, we repeated the phylogenetic regressions for each tree, and summarized the results based solely on whether the effect of population size on genome size was negative (i.e. negative slope) and whether population size was a statistically significant (*P* < 0.05) predictor of genome size (Table 2). We report in Table 2 the test statistics for one randomly chosen phylogeny and include the tallies for regressions using all phylogenies.

**References in the Supplemental Information**

Gregory TR, Nicol JA, Tamm H, Kullman B, Kullman K, Leitch IJ, Murray BG, Kapraun DF, Greilhuber J, Bennett MD. 2007. Eukaryotic genome size databases. *Nucleic Acids Res*. 35(suppl_1):D332-8.

Jones KE et al. 2009. PanTHERIA: a species-level database of life history, ecology, and geography of extant and recently extinct mammals. *Ecology* 90:2648.

Paradis E. & Schliep K. 2019. ape 5.0: an environment for modern phylogenetics and evolutionary analyses in R. *Bioinformatics* 35: 526-528.

Pinheiro J, Bates D, DebRoy S, Sarkar D, R Core Team. 2020. nlme: Linear and nonlinear mixed effects models. R package version 3.1-149.

Upham NS, Esselstyn JA, Jetz W. 2019. Inferring the mammal tree: species-level sets of phylogenies for questions in ecology, evolution, and conservation. *PloS Biology* 17: e3000494.
